# Meta-synthesis of qualitative evidence in road traffic injury prevention: a scoping review of qualitative studies (2000 to 2019)

**DOI:** 10.1186/s13690-020-00493-0

**Published:** 2020-11-03

**Authors:** Saber Azami-Aghdash

**Affiliations:** 1 Research Center for Evidence Based Medicine, Tabriz University of Medical Sciences, Tabriz, Iran; 2 Tabriz Health Services Management Research Center, Health Management and Safety Promotion Research Institute, Tabriz University of Medical Sciences, Tabriz, Iran

**Keywords:** Meta-synthesis, Scoping review, Qualitative study, Road traffic injuries

## Abstract

**Background:**

A considerable number of qualitative studies have been published in recent years on the issues that the quantitative studies have limitations on. This study aimed at performing a meta-synthesis on qualitative studies on Road Traffic Injuries (RTIs) with a scoping review approach.

**Methods:**

This meta-synthesis study was conducted as a scoping review in 2019. The Arkesy and O’Malley framework was applied which has six steps of identifying the research question, identifying the relevant studies, selecting the studies, charting the data, data analysis and reporting the results, and consultation exercise. The required data were gathered by searching the relevant keywords in databases of PubMed, web of knowledge, Scopus, Cochrane Library, Science Direct, Google scholar, Sid, IranMedex. Extracted data were analyzed by the Content-Analysis method.

**Results:**

Finally, 30 studies were included. Extracted data summarized in five main themes and 17 sub-themes. The main themes were: consequences (individual, family, social, financial), the needs of survivors (social support and healthcare), risk factors (general risk factors, risk factors for motorcyclists, risk factors for children and adolescents), barriers of prevention (general barriers, pre-hospital barriers, emergency, and hospital barriers), and prevention solutions (increasing safety, rules and regulations, education, increasing equipment, scientific solutions) of RTIs.

**Conclusion:**

This study combined the methods of the scoping review and the meta-synthesis to mapping all qualitative studies on the RTIs, with this approach, this study provides extensive and practical information for policy-makers, managers, practitioners, and researchers in the field of RTIs. Also, by applying this approach, the gaps in the existing knowledge and areas in need of further research are identified.

**Supplementary Information:**

The online version contains supplementary material available at 10.1186/s13690-020-00493-0.

## Background

Road Traffic Injuries (RTIs) are the main cause of morbidity and mortality in the world nowadays [1]. The biggest proportion of hospital emergency department admissions is comprised of those affected by the RTIs and these admissions result in an enormous amount of direct and indirect costs for both people and the government. So it consumes a considerable share of the country’s annual budget [2]. It is estimated that globally 1.35 million people lose their lives due to RTIs every year and 50 million people get injured [3]. Moreover, it is estimated that these numbers will increase by 65% in the future 20 years [4]. The estimations also show that for each death due to the RTIs, there are 16 cases of hospitalizations and 400 cases of outpatient visits or transient activity limitations [5].

Quantitative studies have been published on various aspects of the RTIs. Although the quantitative studies were brilliant in this area and have helped the prevention of the RTIs, they are faced with some limitations in some aspects. So the researchers used the qualitative methods besides the quantitative ones [6]. The qualitative studies have been the focus of researchers in the field of health sciences in recent years [7, 8]. Despite the successes of the quantitative researches in measurement, analysis, and use of knowledge, they have some limitations in measuring the subjects such as perception, attitude, experience, and feelings of the people. Thus the use of qualitative studies has been grown in fields such as social sciences and health service management [9].

Considering the characteristics of the qualitative studies, in recent years a significant number of these studies have been performed on some aspects of the RTIs that the quantitative studies were faced with serious limitations on those aspects [10, 11]. Summarization of the findings of these qualitative studies may produce some useful information for macro-level policymaking on the RTIs. Thus this study is performed with the aim of meta-synthesis of the qualitative studies on RTIs with the scoping review approach.

## Methods

This was a meta-synthesis study performed as a scoping review in 2019 with the aim of the analysis of published qualitative studies on RTIs. The framework by Arkesy and O’Malley was used which is the first methodological framework to manage the scoping review studies. The framework is published in 2005 and includes six steps: identification of the research question, identification of the relevant studies, selection of the studies, data charting, data analysis and reporting the results, and consultation exercise [12].

- Step one: Identification of the research question

The research question was what are the characteristics and results of the qualitative studies on RTIs. The question is specifically divided into the following:

- What are the main approaches of the qualitative studies on RTIs?
- What are the main methods of data collection in qualitative studies on RTIs?
- What are the most important aspects of RTIs studied in qualitative studies and what are the results?

Inclusion and exclusion criteria: All qualitative studies on the RTIs from January 2000 to March 2019 were eligible to include in the analysis. The language was limited to English and Persian. Those studies on injuries of accidents other than road traffic accidents (such as sailing, aviation, railway), those studies that assessed the RTIs and other injuries at the same time, short communications, and conference abstracts were excluded.

- Step two: Identification of the relevant studies

The required data were gathered by searching the keywords of road traffic injury, road traffic accidents, road traffic crashes, motorcycle accident, motorcycle crash, motorcycle injury, motor vehicle injury, motor vehicle crash, motor vehicle accident, qualitative, interview, phenomenology, focus group discussion, grounded theory at the databases of PubMed, web of knowledge, Scopus, Cochrane Library, Science Direct, Google scholar, Sid, IranMedex (Additional file 1: complete search strategy for PubMed databases). To assure the maximum coverage of the study identified these actions were made: some key journals were hand searched, after removing the irrelevant records the remaining papers were reference checked, they were also citation checked by using the Google Scholar citation, some experts were contacted, and the gray literature was searched through the European Association for Grey Literature Exploitation (EAGLE), the Health Care Management Information Consortium (HMIC), and the System for information on Grey Literature in Europe (SIGLE).

- Step three: Study selection/screening

**Fig. 1:**
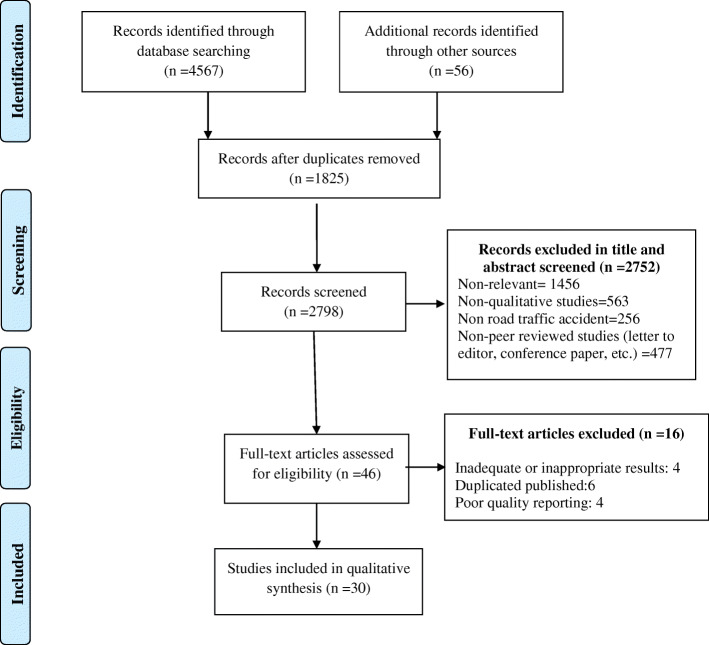
PRISMA chart, screening process of qualitative studies on Road Traffic Injuries (RTIs) published between2000 to 2019

All works of the selection and screening of the papers were performed independently by two members of the research team. Cases of inconsistency between the two were resolved by discussion. Over 80% agreement was the cut of the agreement for the selection and screening of articles between the two researchers. Firstly, the titles of all papers were assessed and those irrelevant to the study purpose were removed. Then the abstracts and full-texts were assessed for eligibility according to inclusion and exclusion criteria. EndNote X5 software was used to handle these works and also to identify the duplications. The Preferred Reporting Items for Systematic Reviews and Meta-Analyses (PRISMA) flow diagram [13] was used to report the findings (Fig. 1).

### Reporting quality assessment

After screening, the reporting quality of the studies was assessed by two researchers using the Critical Appraisal Skills Program (CASP) checklist. The checklist includes 10 items. The first two items are screening questions. The appraisal of the study would continue only if the answer to at least one of these two questions was yes. For the next eight questions the three options of Yes, Can’t tell, No were marked for which the scores of three, two, one were assigned respectively [14]. So the maximum score of each paper was 24 and the minimum was eight. The inconsistencies were resolved by discussion.

- Step four: Categorization of the data

To extract the data, the data extraction form was developed in MS Word 2010. Data for three papers were extracted as a pilot. Then the form was revised. The intended data included: author(s), year, country, study purpose, participants, the approach of the study, data collection method, study findings (themes and sub-themes).

- Step five: Conclusion, summarization, and reporting the results

The gathered data were analyzed by the content analysis method. Content-Analysis is a widespread method for the analysis of qualitative data through the identification, analysis, and reporting of the patterns (themes) within a text [15–18]. Coding the text was performed by two researchers independently. Steps of the analysis were: getting familiarized with data, identifying primary areas, putting the paper in the areas, reviewing the papers of each area to complete the findings, assuring the reliability of the work by comparing the results by the two coders.

- Step six: Providing practical recommendations

After extracting and reporting the study results, based on the study findings and the opinions of the research team, practical recommendations were made in terms of research methodology and also for the policymakers and managers.

## Results

Of the 4623 retrieved records, 1825 were duplicates. At the title and abstract screening, 2752 records were removed. The full-text review also resulted in the removal of 16 papers so finally, 30 papers were included in the synthesis (Fig. 1).

The characteristics of the included studies are shown in Table 1. They are conducted in 12 countries most of which (nine countries) are low-and-middle-income countries (LMICs). The total number of the participants of the included studies was 906 people. The interview was used in 25 studies, FGD in eight studies, and the nominal group in one study (four studies had used more than one method) as a data collection method. The approach of the study was not mentioned in 12 studies. Seven studies have used content analysis, six phenomenologies, and five grounded theory.

**Table 1:** Characteristic and results of qualitative studies on Road Traffic Injuries (RTIs) published between2000 to 2019 (*N* = 30)

Author, year	Country	Aim of study	Participants (number)	Approach	Data collection	Results	
						Main Categories	Subcategories
Doohan and Saveman, 2014 [19]	Sweden	nonphysical consequences of a multifatality bus crash and the subsequent effect on the surviving passengers’ lives	survivors of a major bus crash (56)	NM	IDIs	Reacting to the crash	Feeling, thinking and helping others
							Reacting to the emergency care
							Encountering the Media
							Receiving formal support
						Processing the crash	Healing with social Support
							Difficulties when sleeping
							Everyday travelling
							Seeking closure
Pashaei Sabet et al., 2016 [20]	Iran	experiences of encountering with physical trauma resulting from traffic accidents	participants had a record of upper and or lower extremity injuries caused by traffic accidents (NM)	CA	IDIs	experiencing some limitations	1. Limitations in daily activities
							2. Dependency
						disturbances in performing professional duties	–
						family problems caused by trauma	–
Yadav and Shrestha, 2017 [21]	Nepal	experience of oral and maxillofacial trauma patients due to road traffic accident right from immediate after the accident till the end of definitive treatment	oral and maxillofacial trauma patients due to road traffic accident (20)	Phenomenology	IDIs	unreal experiences	
						emotional responses	
						need to inform and need for information	
						need for assistance	
						perception toward the maxillofacial injury	
						experience on treatment	
						staff-patient interaction	
Franzen et al., 2006 [22]	Sweden	experiences of pre-hospital and hospital care and subsequent rehabilitation	people injured in a traffic environment (9)	NM	IDIs	Facing commotion	Feeling uncomfortable due to memory loss
							Feeling embarrassed
						Experiencing trust and security	Being the centre of attention
							Having confidence in caregivers and relatives
						Lacking security and support	Feeling worried and uncertain
							Feeling neglected and disrespected
							Feeling hindered
						Struggling to return to everyday life	1. Longing for daily routines
							2. Doubting the will to become healthy
							3. Finding ways to cope with pain
Pashaei Sabet et al., 2014 [23]	Iran	understand the rehabilitation needs of patients with physical disabilities from road traffic accidents to return to the community	both genders and age ranged between 18 and 45 years old with at least 3 months physical disability in upper and lower limbs or spinal cord injury (12)	CA	IDIs	the need to be under the umbrella of support	Need for support by the care team
							The need for social support
							Tendency to spirituality
						the need for continuity of care	Liberation in society
							Caring knowledge search
						achieving independence	–
Ghorashi et al., 2012 [24]	Iran	reasons of motorcycle accidents	motorcycle drivers in streets, injured motorcycle drivers in hospital and their families, old car drivers, traffic wardens and nurses (17)	CA	IDIs	motorcycle as entertainment tool	alcohol and drug abuse
							racing
							showing
						environmental and technical factors	1. not adhere to laws
							2. challenge with police
							3. escaping of helmet
							4. purchasing power
						economic and cultural factors	ill-favored climate
							Impaired motorcycle
							neglecting motorcycles
Batool et al., 2012 [25]	Pakistan	road safety issues	government officials, academics and the general driving population (31)	NM	IDIs	Institutional Issues	Low valuation of road safety
							Institutional Weaknesses
						Execution Issues	Lack of human resources
							Lack of timely implementation
						Physical &Operational Issues	Increasing motorization and urbanization
							Traffic mix on roads
							Non-standardized driving practices
							Poor public transport system
							On-road encroachments and capacity issues
							Out-dated traffic management
							poor licensing and penalties system
							Social injustice
							Poor roads and vehicle maintenance standards
						Attitudinal and behavioural issues	Characteristics of unsafe drivers
							attitudinal and behavioural problems
							Societal and cultural issues
						Road safety research and accident data bank	Poor accident reporting and recording system
							Absence of comprehensive data bank
							Inaccessible and inadequate dissemination of research work
							Reliance on old research work
Christie et al., 2007 [26]	UK	children’s exposure to road traffic injury risk in low socioeconomic areas	Parents of children aged 9–14 years living in low socioeconomic areas (86)	NM	FGDs	Hazards caused by drivers and riders	
						Insufficient parental responsibility	
						Risk taking by children	
						Lack of activities and facilities	
						Parents’ views on solutions	
Sanusi and Emmelin, 2015 [27]	Nigeria	risk and road safety as well as of protective measures	commercial motorcycle driver’s (10)	NM	IDIs	Risk-taking as generally acceptable	Inadequate training and licensing
							Poor law enforcement
							Risk-saturated environment
						Risk-taking as an intrinsic part of the occupation	Profit based on overriding rules
							Assumptions of safety
							Unavoidable accidents
							Constant exposure
						Risk-taking as a way to make ends meet	A fight to feed and survive
							Family responsibilities
							Unaffordable safety measures
Tetali et al., 2013 [28]	India	perceptions of stakeholders on road safety	government officials, subject experts, and road traffic injury victims, trauma surgeons, medical interns, nurses, and taxi drivers (37)	NM	IDIs and FGDs	Status of road safety	1. Unsafe roads
						Law and enforcement	1. Ineffective enforcement
							2. Unequal enforcement
							3. Lack of political-will
							4. Fines are not a deterrent
							5. Corruption
							6. Low compliance
							7. Disregard of rules
						Road engineering	1. Poor roads
							2. Poor infrastructure
							3. Poor road-use
						High-risk road users	1. Passenger-seeking attitude
							2. Media influence
							3. Lack of parental control
							4. Thrill seeking
						Responsibility for road safety	1. Individual responsibility
							2. Government’s responsibility
							3. Collective responsibility
						Strategies to improve road safety in Hyderabad	1. Awareness generation
							2. Enforcement
							3. Non-economic penalties
							4. Stricter penalties
Hashemiparast et al., 2017a [29]	Iran	Explore the reasons for risky road crossing behaviors among young people.	males and females who had a car-accident (12)	CA	IDIs	Conformity with the masses	1. conformity with peers
							2. conformity with the public space of society
						anomie	–
Shams et al., 2010 [30]	Iran	views of taxi drivers about risky driving behaviors	taxi drivers (42)	NM	FGDs	the role of taxi drivers in current driving situation	–
						drivers’ reasons for committing risky driving behaviors	Behavioral reasons
							Non-Behavioral reasons
						actions for modifying risky driving behaviors	Suitable education
							Monitoring for roles
							Correcting the streets
							Providing suitable facilities for driving
							Resolving Community Structural Problems
							Carry out hazardous driving behaviors modification interventions
						suitable places for implementing the recommended interventions	Drivers’ gathering places
							Interior Space and Taxi Body
							Taxi Drivers’ Routes
						best channels for communicating and persuading taxi drivers	Mass media
							Writing media
							Effective people on the behavior of taxi drivers
Zamani-Alavijeh et al., 2010 [31]	Iran	explore risk behaviors among Iranian motorcyclists	Motorcyclists (32)	GT	IDIs and FGDs	personal characteristics	Individual features
							Physical and mental health and balance
							Knowledge and skill
							Motivation to use motorcycleThe reaction of the individual to previous experiences and behaviors
						Social factors	Police performance
							low cost and easy availability of motorcycles
							motorcycle defects and land ownership laws
							traffic laws
							traffic culture
						vehicle related factors	Type of motorcycle
							Motorcycle breakdown
						abuse of safe equipment’s	1. Motorcycle Safety Equipment
							2. Motorcyclist Safety Equipment
						environmental factors	Type and structure of roads
							lack of special motorcycle route
							road safetyair condition
Khorasani-Zavareh et al., 2009 [32]	Iran	barriers effective post-crash management	medical services personnel, police officers, members of Red Crescent, firefighters, public-health professionals, road administrators; some road users and traffic injury victims (36)	GT	IDIs	involvement of laypeople	1. Cultural background
							2. limitations in knowledge
							3. late arrival of the emergency services
						lack of coordination	lack of a systematic approach
							different ambulance dispatch site locations
							existence of parallel organizations with the same activity
							substandard telecommunication equipment
							undeveloped satellite navigation
						inadequate pre-hospital services	low number of ambulance dispatch sites
							inadequate human resources
							insufficient physical resources
							lack of police officers
							lack of crash scene management skills
						shortcomings in infrastructure	1. poor urban infrastructure
							2. no satellite navigation
Haghparast-Bidgoli et al., 2013 [33]	Iran	influencing an effective trauma care delivery at emergency departments (EDs)	health professionals (15) and injured patients (20)	GT	IDIs	Inappropriate structure of hospitals	Teaching hospitals
							Inappropriate layout and planning of ED premises
						Unsupportive environment	Absence of established ways and inappropriate facilities for communication
							An environment of mistrust
							Low economic incentives
						Shortage of staff	–
						Unclear national policies	Absence of an established trauma system
							Lack of continuity between pre-hospital and hospital trauma care processes
						Poor organization of care at the ED	1. Absence of established trauma teams
							2. Lack of protocols and guidelines for trauma care
							3. Inappropriate human resource planning
Haghparast-Bidgoli et al., 2010 [34]	Iran	explore prehospital trauma care process for RTI victims	pre-hospital trauma care professionals (15)	GT	IDIs	administration and organization	Inappropriate management
							inefficient structure
							inefficient rules and regulation
						staff qualifications and competences	Inappropriate training plans
							out of date, unpractical and inadequate training courses
						availability and distribution of resources	Deficiency of resources
							misdistribution of resources
						communication and transportation	inappropriate communication system
							ineffective medical direction and referral system
						involved organizations	poor coordination and cooperation between organizations
							insufficient knowledge and skills regarding the rescue of victims
							insufficient knowledge and skills regarding managing the crash
						laypeople	Providing incomplete or wrong information
							emotional reactions
							conflicts with the EMS personnel
						infrastructure	lack of GPS system
							sub-standard road infrastructures
							lack of infrastructures for helicopter ambulances in the big cities
							an inadequate telecommunication system
Alinia et al., 2015 [35]	Iran	explore the barriers of pre-hospital care in traffic injuries	Peoples with at least 2 years’ experience in the field of pre-hospital services (18)	CA	IDIs	people	Inadequate knowledge about first aids
							Laypeople -Involvement
							Mistake calls
						Metropolitan infrastructure	Traffic
							Accessibility to streets and alleys
							Naming of alleys
						profession	Professional Autonomy
							Workload
							Work-related injuries
						managerial issues	1. Inadequate telecommunication technology
							2. Inadequate human resources
							3. Inappropriate workload related privilege
							4. Lack of organizational coordination
Razzaghi et al., 2017 [36]	Iran	explore the obstacles relating to the elderly pedestrians	elderly pedestrians age equal or more than 60 years old (23)	CA	IDIs	Problems related to environment	
						Social respect to elderly	
						physical health	
Hashemiparast et al., 2017b [37]	Iran	explore the young pedestrians risky road crossing behaviors reasons	young individuals who had the experience of vehicle-collision accident (12)	CA	IDIs	conformity with the masses/crowds	
						bypassing the law/ law evasion	
						lack of social cohesion and sense of belonging in social relations’	
Perez-Nunez et al., 2012 [38]	Mexico	consequences of fatal and non-fatal road traffic injuries	injured and relatives of people who died (24)	Phenomenology	IDIs	Health Consequences	depend on others to perform the activities of daily life
							Changes in family members health during the care of injured people
							mental health
							feelings of sadness and pain
						Consequences on family life	change of roles
							change of family composition
						Household effects associated to the monetary cost	Expenditures associated to RTI
							Leave households without money to bury
							Families change even their eating habits
							Households lose services
							Injured people and some of their relatives stop working
							Role change
							Loss of capital
							Loss of a provider
							Loss of personal and household’s patrimony
Noori Hekmat et al., 2015 [39]	Iran	explore the challenges and complexities related to health care financing for traffic victims	managers at the Ministry of Health, Medical Sciences Universities, trauma specialized hospitals and basic insurances (36)	Phenomenology	IDIs	financial integration	Lack of timely payment of contributions to the Ministry of Health
							Injustice in aggregating financial resources
							The complex process of aggregating financial resources
						accumulation	Lack of legal authority for economic activity
							Financial instability
							Lack of fair distribution of financial resources
							The complex and timely process of allocating and distributing financial resources
							Challenge and tension over deductions
						distribution of financial resources and service purchasing	Non-transparency of the criteria for identifying the injured
							Lack of Comprehensive Coverage for Service Article 92
							Defective service coverage
							Therapeutic Services Package
							Lack of number of trauma services providers
							General challenges Buy service
Bazeli et al., 2017 [40]	Iran	explore the challenges and facilitators in management of mass casualty traffic incidents	experienced managers, paramedics and staff of aid organizations (14)	GT	IDIs	Multiplicity of relief agencies	Several organizations are involved in managing these events.
							The accident scene is managed by several organizations.
							In most accidents involve at least three or four of emergency agencies
						Lack of clear roles	Limit set of organizational tasks is not defined.
							There is no clear description of personnel’s job
						Lake of centralized and integrated commanding	There is no a certain Commander
							Currently we do not have a unified command
						Cultural factors	Citizens do not adhere traffic rules
							traditional management culture
Huicho et al., 2012 [41]	Peru	assess current interventions implemented to reduce RTIs	policymakers and technical officers involved (19)	NM	IDIs	Lack of clear and sustained political and budgetary support	
						Ineffective coordination between the different sectors involved	
						Insufficient community participation	
						Lack a reliable and fully functional information system	
Ramos et al., 2008 [42]	Spain	Young people’s perceptions of traffic injury risks, prevention and enforcement measures:	Informants (43) and Young people (98)	NM	IDIs & FGDs	Determinants of traffic injuries	**personal**
							drug use
							false sense of security which comes from well equipped cars
							enjoyment of the sensation of speed
							distractions (using cell-phones, reading the newspaper or arguing while driving)
							fatigue
							night driving
							being male
							low educational level
							**social**
							1. rebelliousness of youth against norms
							2. permissiveness of Mediterranean culture about drug use
							3. having parents who break rules as a model
							4. the social value attributed to vehicles as symbols of freedom
							5. the early age at which moped driving is allowed
							6. job-pressure on professional drivers
							7. the lack of public transport
							**structural**
							unsuitable design of roads
							siting of clubs far away from towns
							rising traffic densities
						Relevance and trends in traffic injuries	important problem
							leading cause of death
							complex problem to tackle
							RTIs injuries and deaths are avoidable
							Traffic injuries are declining
						Driving while under the influence of psychoactive substances	
						Assessment of interventions which are carried out	quite ineffective
							applied too late
						Intervention proposals	improve public transport
							sanctions and incentives
							measures to reduce adverse effects of drugs on driving
							design cities more suited for pedestrians
							generate social debate
Soori et al., 2015 [43]	Iran	Opportunities and barriers to enacting mandatory child car restraint laws in Iran	road safety stakeholders (28)	Phenomenology	FGDs	Barriers and threats	Lack of propaganda by mass media
							Lack of related laws
							Lack of parents’ awareness
							Lack of a positive attitude among households
							It is not a priority for the children’s needs
							Lack of accessibility
							It is too expensive to purchase for everyone
							It is hard to find it in the market
							Policy-makers do not know about its benefits
							Children dislike to use it
						Opportunities and facilities	Family sensitivity to their children’s health
							Officials’ supports
							National facilities
							Executive facilities of traffic police
							support of relevant organisations
							Possibility to mass production by domestic industries
Trevino-Siller et al., 2011 [44]	Mexico	prioritise road traffic injury (RTI) interventions	road users and social groups (48)	NM	FGDs, NGs	1. Massive educational campaign	
						2. Vital education programmes in schools	
						3. Increase and improve streets and avenues	
						4. Urban planning policies to locate schools and parking lots	
						5. Obligatory programmes for bars	
						6. Special paths for pedestrian crossings in risk zones	
						7. Clear signalization for pedestrians and bus stops	
						8. Obligatory exam to obtain drivers license and increase minimum age for drivers	
						9. Implementation of punitive law system	
						10. Permanent program for selection and training of policemen and salaries improvement	
Ainy et al., 2011 [45]	Iran	Presenting a practical model for governmental political mapping on road traffic injuries	experts from governmental and non-governmental organizations (26)	Phenomenology	FGDs	suggested organization to be the leading agency in prevention of RTIs	Traffic police
							Presidential institution
							Ministry of interior
							Parliament
							Cabinet
							Ministry of roads and transport
							Judiciary
						suggestions proposed to resolve the inadequacies of planning and prevention of RTIs	Correction related laws and determine the duties
							Credit allocation necessary
							Careful planning and coherent
							Sensitive authorities
							Unique management
							Doing Research to achieve accurate statistics
							Determine organization responsible for
Salari et al., 2017 [46]	Iran	explore strategies to control RTIs	Mainly representatives from the police, Ministry of Road, Municipal, emergency services and Minis-try of Health (30)	NM	IDIs	Accident scene management	Integration
							The use of a single Relay phone Number
							Scientific examination of causes of accident
						Governance and Leadership	Establishing a leading agency responsible for RTIs
						Improving Accident database	Integrated Database
						Education	public education, and creation of awareness
						Ensuring safe driving by Enforcement	random testing of the use of alcohol and drugs
							Increasing fines for traffic violations
							Increasing the number of speed cameras
						Ensuring safe driving by restriction	Instituting Psychological examination as part of the tests to acquire driver’s license
							Restricting teens from driving at night
						Ensuring the safety of Pedestrians	Construction of pedestrian bridges/overpass
Patel et al.:2017 [47]	Brazil	causes of delays in pre-hospital transport of RTIs patients	health care providers employed at prehospital or hospital settings (11)	Phenomenology	IDIs	Traffic related issues	High traffic volume
							Wrong navigation information
						Lack of public education	1. Lack of traffic education
							2. Lack of public education to respond to trauma
							3. Lack of drivers awareness of ambulance right-of-way
						Insufficient personnel	Lack of personnel
						Poor location	Stations distance from crashes sites
							Stations located far from important places of the city
						Insufficient ambulances	Lack of equipment in the ambulances
							Not enough ambulances
						Bureaucracy	1. Long time to receive notification within the pre-hospital care system
							2. Difficulty with patient admission
Teye-Kwadjo et al.:2017 [48]	Ghana	risk factors for road transport-related injury among pedestrians	Pedestrians (26)	NM	IDIs	Behavioral factors	1. Pedestrians share lanes with vehicles
							2. Pedestrians’ non-use of visibility aids at night
							3. Walking with face turned against traffic
							4. Cell phone use while walking
							5. Drivers not yielding of right-of-way
							6. Speeding
							7. Repeated honking
							8. Distracted driving (driver chatting with front
							9. seat vehicle occupants)
							10. Roadside trading/trading in motorised traffic
						Personal factors	Low pedestrian crash risk perception
							Pedestrian and driver attitudes to right-of way laws
							Inaccurate vehicle speed estimation
							Inconsiderate driving attitudes
						Environmental factors	1. Narrow roadways
							2. No sidewalk and crosswalks
							3. No footbridges
							4. Unsignalised zebra crossings
							5. Nearness of food joints to roadways
							6. Uncovered roadside drains
							7. Nonexistent speed limits on community roads

*IDIs* In-depth Interviewees, *FGDs* Focus Group Discussion, *CA* Content-Analyze, *GT* Grounded Theory, *NGs* Nominal Groups

**Fig. 2:**
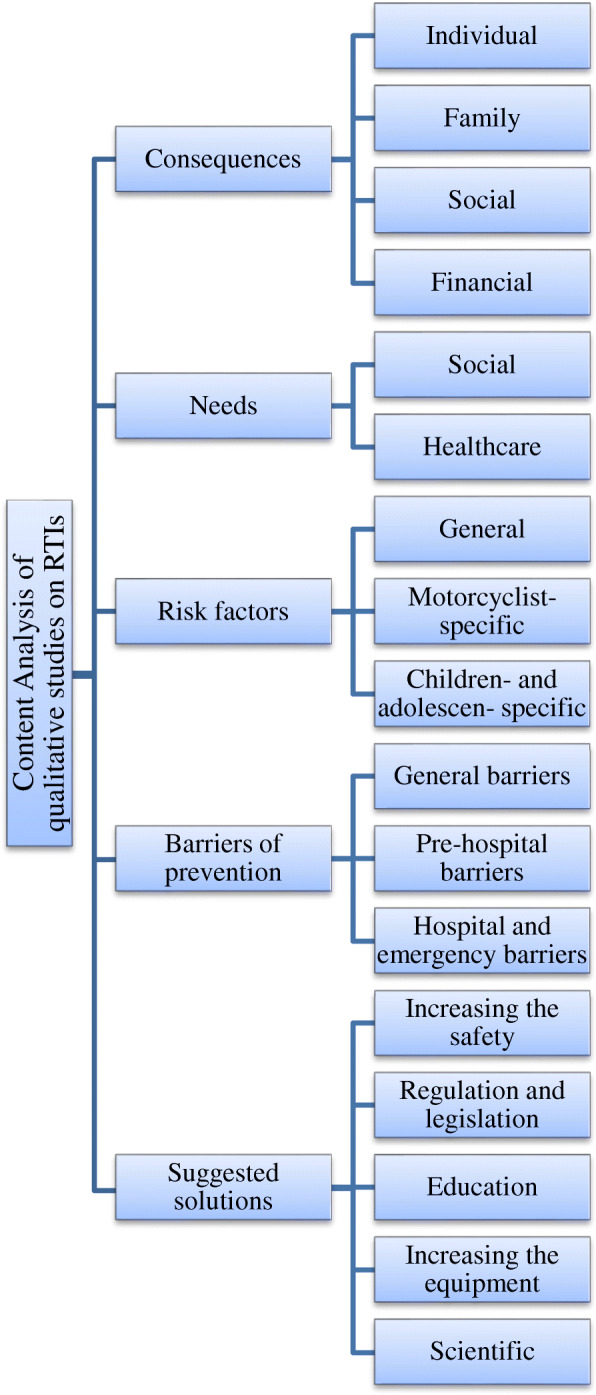
Mapping the extracted results from qualitative studies on Road Traffic Injuries (RTIs) published between2000 to 2019

Extracted data were summarized into five main themes and 17 sub-themes by the content-analysis (Fig. 2).

### Consequences

The consequences of the RTIs were divided into four categories of individual, family, social, and financial consequences.

#### Individual consequences

One main individual consequence of the RTIs was the physical so that even if the individual survives at the accident, he/she will suffer from morbidity. Long-term pains, movement problems, and sleep problems were major examples of physical problems. Beyond the mortality and the morbidity, some mental problems also occur as a result of the RTIs such as feeling ashamed, being an encumbrance, and fear of the future.

#### Family consequences

Further to the individual consequences, the RTIs also have consequences on families. The major problems in this category were problems of caring for the injured people (skills, costs, the stamina of caring), change in roles of the family members such as the breadwinner role of the mother of children due to injury of the father), and cut or reduction of family income.

#### Social consequences

One main social consequence of the RTIs that was highly mentioned was the limitations of the social relations of the injured people. Moreover, the accidents due to the low safety of the vehicles and roads can result in distrust of the people in government actions.

#### Financial consequences

One of the most obvious consequences of RTIs is financial consequences. It includes damages to the vehicle, damages to road facilities, treatment and care costs of the injured people, costs of losing the productivity of the people in the society, paying the blood money, and other costs.

### Needs of survivors

Every RTIs due to the mentioned consequences creates some needs in the injured individual and his/her family. One of these needs is the social needs of the injured people which include social support by the government, charities, family, and friends in terms of financial, mental, spiritual, and legal supports. Another need after an RTI is the need for healthcare which includes emergency care right after the accident, specialist and quality care at the hospital, rehabilitation care, and mental care.

### Risk factors

The other main theme was the risk factors of the RTIs which had three sub-themes of general risk factors (five items), risk factors for motorcyclists (five items), risk factors for children and adolescents (four items). Table 2 shows the risk factors of the RTIs.

**Table 2:** Risk factors of the Road Traffic Injuries (RTIs) derived from the qualitative studies published between 2000 to 2019

Sub-theme	Risk factors
General risk factors	1. Inadequate and ineffective training before getting licensed
	2. Poor supervision (shortages of manpower, limited facilities, and no rigid supervision)
	3. Unsafe environment for driving (Unsafe roads, Unsafe vehicles)
	4. Lack of responsibility and contribution of the families
	5. Risky behaviors of the drivers (drunk driving, high speed, using a cellphone, and so on)
Risk factors of motorcyclists	1. Risky behaviors of motorcyclists (show plays, drunk riding, no use of helmet, using a cellphone, and so on)
	2. The low purchasing power of the motorcyclists to buy a proper motorcycle, helmet, and other safety tools (remind that most studies were conducted at LMICs)
	3. Limitations in developing and executing the legislation related to motorcyclists
	4. Lack of special ways for motorcyclists
	5. The vulnerability of the motorcycles in adverse weather conditions
Risk factors of children and adolescents	1. Risk factors related to the parents (Low use of specific safety equipment, insufficient care of the children, law-breaking by parents and then becoming a model by children and adolescents, and so on)
	2. Risk factors related to the children and adolescents (enjoying high speed, low ability of analysis and understanding, and so on)
	3. Impact of peers
	4. Low knowledge of rules, regulations, and safety rules

### Barriers to prevention

The barriers to the prevention of the RTIs were in three categories of general barriers (five main and 13 sub-main), pre-hospital barriers (three main and 10 sub-main), emergency, and hospital barriers (two main and five sub-main). Table 3 shows the themes and sub-themes of the barriers to the prevention of the RTIs.

**Table 3:** Barriers to prevent the Road Traffic Injuries (RTIs) and reduce their consequences according to qualitative studies published between 2000 to 2019

Dimension	Main barriers	Secondary barriers
**General barriers**	Organizational	1. Low priority of the RTIs in government agenda
		2. Weaknesses in organizing
		3. Lack of sufficient support and political commitment for the prevention of the RTIs
	Administrative	1. Shortages of manpower
		2. Poor road safety standards
	Socio-cultural	1. Social inequity
		2. Behavioral and believe problems
		3. Undefined and little role of the people in the prevention of the RTIs
	Scientific	1. Lack of an integrated and effective registration and reporting system
		2. Limitations in applicable researches
	Systemic	1. Poor public transportation
		2. Traditional management (lack of scientific and systemic attitude in the management of traffic accidents)
		3. Problems and weaknesses of driving license system
**Pre-hospital barriers**	General public	1. Low knowledge and wrong interventions at the accident scene
		2. Making traffic jams for relief forces
		3. Low culture (harassing phone calls to road EMS)
	Coordination	1. Lack of an integrated and orderly system
		2. Lack of a leading and steward organization with sufficient facilities and authorities
		3. Shortages of communication and coordination equipment
		4. Weaknesses of regulations and laws
	Limited facilities	1. A limited number of ambulances, equipment, and facilities
		2. Shortage of capable manpower and inappropriate distribution of them
		3. Low use of air aid
**Emergency and hospital barriers**	Poor organization	1. Most hospitals are educational (care by students with low knowledge and experience)
		2. Poor planning and control of the emergency departments
		3. Shortages of care guidelines of trauma patients
	Manpower	1. Shortage of capable manpower in the care of trauma patients
		2. Poor planning and management of the manpower

### Solutions of prevention

Solutions for prevention of the RTIs and reduction of their consequences were categorized in five dimensions including increasing safety (three items), rules and regulations (four items), education (two items), increasing equipment (five items), and scientific solutions (two items). Table 4 shows these solutions.

**Table 4:** Solutions of prevention of the RTIs and reduction of their consequences according to the qualitative studies

Dimensions	Solutions
**Increasing safety**	1. Increasing the safety of the roads and vehicles
	2. Promotion of the use of safety equipment (seat belt, helmet, …)
	3. Making specific ways for bike riders and motorcyclists
**Rules and regulations**	1. Preventing drunk driving
	2. More rigidity in driving licensing
	3. Appropriate penalties for violation of the regulations
	4. Making a leader and steward organization with sufficient facilities and authorities
**Education**	1. Public education by mass media such as TV
	2. Specific education at schools
**Increasing equipment**	1. Increasing public transportation
	2. Increasing the human resources for prevention of the RTIs
	3. Providing sufficient support and finance
	4. Use of new safety tools (intelligent road cameras, driver control cameras, …)
	5. Strengthening pre-hospital EMS
**Scientific solutions**	1. Effective long-term planning
	2. Implementing effective and in-time registration, reporting system, and applicable researches

Results of the quality appraisal of the included qualitative studies showed that the average quality score of them was 21.6 in the 8–24 range. One issue that did not receive sufficient attention in the studies was the ethical issues (Additional file 2).

## Discussion

Of the 4623 retrieved papers, finally, 30 included in the study. The synthesis of the qualitative data resulted in five main themes and 17 sub-themes. The themes were consequences of the RTIs (individual, family, social, financial), needs (social support, healthcare), risk factors of the RTIs (five general risk factors, five risk factors of motorcyclists, four risk factors of children and adolescents), barriers of prevention of the RTIs (five general barriers, three pre-hospital barriers, two hospitals, and emergency barriers), and solutions of prevention of the RTIs (three items on increasing safety, four items on regulation, two items on education, five items on increasing equipment, and two items of scientific solutions).

### Consequences of the RTIs

The RTIs not only cause physical and financial problems, but also cause some mental problems due to losing family members, feeling guilty, feeling ashamed, being an encumbrance, and fear of the future. The physical problems usually get better by healthcare or the person becomes adapted to the problems. But the mental problems such as long-term depressions bring more suffering for the person and have more severe consequences [49]. A meta-analysis by Wanli Lin and colleagues (2018) showed that the prevalence of Post-Traumatic Stress Disorder (PTSD) among 6804 victims of the RTIs was 22.2% [50]. Another meta-analysis by Dai et al. (2018) on 1532 children and adolescents injured in RTIs showed the prevalence of PTSD as 19.9% [51]. The study by Asuquo et al. in Nigeria (2017) showed that 63% of the victims of the RTIs became depressed [52]. Some other studies also indicated the high prevalence of mental problems among injured people in the RTIs [53–57]. Thus the mental problems of these people should be considered to provide appropriate care.

Another consequence of RTIs is financial issues. The high social and economic costs of the RTIs have challenged the policymakers of the countries [38, 58]. The economic costs of the RTIs include all costs of the RTIs and costs due to the RTIs [59]. It is estimated that the global costs of the RTIs be US $ 518 billion of which the US $ 65 billion is at the LMICs. It is also estimated that the costs of the RTIs at the low-income, middle-income, and high-income countries to be 1, 1.5, and 2% of the Gross Domestic Product (GDP) of that country, respectively [4]. According to the study by Eyni et al. (2014) which applied the willingness to pay (WTP) method found that the costs of the RTIs equal to 6.46% of the GDP of Iran [60]. A glance at the literature shows that several methods have been used to estimate the costs of the RTIs in recent years such as life insurance approach, court award, compensation method, implicit public sector valuation, gross output, Human Capital (HC), Willingness To Pay (WTP) [58, 61–65]. The systematic review by Azami-aghdash et al. (2018) showed that the HC method is more frequently used for this purpose [66].

### Needs of survivors

One of the main needs of the victims of the RTIs is the need for social support because they cause the victims to be socially isolated [67, 68]. Numerous studies have shown that good social support to the survivors of the RTIs helps them to get better quickly and to overcome mental problems [69–72]. Family, friends, and some peer groups in the society can provide good social support for these people [73, 74]. The important point to consider in this regard is that to get the most possible impact, the support should be according to the conditions of the injured one and his/her injury.

### Risk factors

One of the most important categories of the risk factors identified in this study is the risk factors of the motorcyclists. According to the National Highway Traffic Safety Administration (NHTSA), the risk of death of the motorcyclists is 34 times more than other vehicles. This number is eight times for severe injuries [75]. Most of the studies in this review also indicated a higher risk of injury to the motorcyclists [76–80]. It seems that the prevalence of using motorcycles has been grown so fast that the culture of its proper use has been lagged. So that people are not familiar with the culture of right and safe use of motorcycles [81]. Another reason for the higher rate of injuries of the motorcyclists might be its lower safety equipment compared to other vehicles [82, 83]. Moreover, compared to the other vehicles, most of the users of the motorcycles are the youth and peoples at this age due to the nature of the age and the more tendencies for excitement are at the higher risk of accident [84]. So, safer design of the motorcycles and more preventive laws along with the more measures to promote helmet seem necessary. Six risk factors were identified for the motorcyclists in this study. Quantitative studies have identified numerous risk factors for the motorcyclists [85–89]. Since in this study the risk factors are identified from qualitative studies, merging the findings of the qualitative and quantitative studies may provide a broader view on the issue.

As it is mentioned, the risk factors identified from qualitative papers in this study were general risk factors, specifically for motorcyclists and children and adolescents. Yet quantitative studies have identified specific risk factors for other groups of people such as the elderly, pedestrians, and bike riders. The literature shows that these groups are also vulnerable to RTIs [90–96] and should be investigated by qualitative studies.

### Barriers to prevention

The study by Khorasani Zavareh et al. (2009) showed that there are several barriers to the prevention of the RTIs in Iran. The main theme of the study was the lack of a systemic approach to the prevention of the RTIs and the sub-themes were human resources, transportation systems, and organizational coordination [97]. A report by Hyder et al. (2013) assessed the barriers to prevention of the RTIs including knowledge, attitude, participation, management, capacity building, and infrastructure in five dimensions of government, health sector, society, academics, and private sector [98]. Alinia and colleagues (2015) studied the barriers of providing pre-hospital EMS care for the RTI victims and found 13 barriers in 4 main areas of barriers related to people, barriers related to the structure of the metropolises, barriers related to professions, and managerial barriers [35]. This study found few barriers in hospital and hospital emergency departments which might be mainly due to a limited number of studies in this regard. But other studies have shown that the hospital emergency medical care has a significant role in reducing the mortality and morbidity due to the RTIs [99, 100]. Thus it is suggested that more qualitative studies be conducted on the barriers to providing quality care for the RTI victims at the hospital emergency departments.

### Solutions of prevention

The existence of a leading organization with sufficient authority and tools is one of the most important solutions for the prevention of the RTIs. Several organizations are involved in RTIs and their prevention, of which the main ones are the ministry of transportation, ministry of industry, ministry of health, traffic Police, forensic medicine organization, Central insurance organization, ministry of Justice, ministry of interior, the red crescent organization, and the EMS. At the countries that are successful in reducing the burden of the RTIs, usually, there is a leading organization that has the stewardship of the activities around the RTIs [101, 102]. For example, in Canada, the federal and provincial governments are the pioneer of road safety. The federal government has a commanding role in the transportation system and participates in the transportation system development by data collection and research. The police have the administrative role and develop safety plans with the help of the Judiciary [103]. The study by Soori et al. (2009) proposed the Traffic Police or the president as the steward leader in the prevention of the RTIs in Iran [104].

Another important solution that was emphasized in several studies is establishing an on-time and effective registration and reporting system. The experiences of the countries indicate that the health sector can play an effective role in designing and implementing the recording and reporting system of the RTIs [105–108]. In India, for example, the project named “Road Traffic Injury Surveillance Project” The project implemented in 2007 by the Indian Council of Medical Research Association (ICMRA), World Health Organization (WHO), ministry of health and family welfare after the many problems of the health sector data system. The main purpose of the project was to establish a care system in 25 major hospitals of India which then achieved considerable successes [109]. The system then merged with the Integrated Disease Surveillance Project (IDSP) by the government [110]. The other example is the case of Pakistan in which the health system of the country developed and implemented the RTIs’ care system in 2006. The goal of the system was to estimate the burden of the RTIs, to study the RTIs’ victims admitted to the hospital, and to provide solutions for reducing the RTIs. As a result of implementing this system, the attention to the RTIs was increased and the outputs of the system showed that the real number of the victims of the RTIs is higher than the statistics by the police [111]. The point to keep in mind is the cooperation of the health sector with other sectors and organizations in designing and implementing such systems. Since the RTIs are multilateral and many organizations are involved in it, the data of the RTIs should be integrated from all involved organizations.

### Study limitation

Although with the best of our knowledge this is the first study of scoping review and meta-synthesis on qualitative studies on the RTIs, it has some limitations. The main limitation of this study was limiting the search of the literature to English and Persian languages because there might be some good studies in other languages that are not included in the synthesis. Subjective interpretation of the findings is another limitation of this study. Petticrew et al. (2013) noted that the results of meta-synthesis should be more interpreted by policy-makers and users [112]. But in the present study, this was not possible.

## Conclusion

This study combined the methods of the scoping review and the meta-synthesis to mapping all qualitative studies on the RTIs to summarize the vast literature into five main themes and 17 sub-themes. The main themes found in this study were: consequences of the RTIs, needs, risk factors, barriers of prevention, and solutions for the prevention of the RTIs. With this approach, this study provides extensive and practical information for policy-makers, managers, practitioners, and researchers in the field of RTIs. Also, by applying this approach, the gaps in the existing knowledge and areas in need of further research are identified. However, this method is a new method and more studies are needed to become more mature with this method.

### Future research

Based on the results of this study, the following topics are recommended for future qualitative studies:

- ➢ Psychological and social effects of traffic accidents.
- ➢ Policy-making, management, and organizational tasks of RTIs prevention.
- ➢ Qualitative studies to investigate RTIs prevention issues in high-risk and vulnerable groups (elderly, children, disabled people, etc.).
- ➢ Qualitative studies to further investigate the provision of high-quality health care services to traffic accident victims
- ➢ Carrying out qualitative studies on experiences, high-risk behaviors, and prevention of traffic accidents with the participation of drivers of public and heavy vehicles
- ➢ A qualitative study with policy-makers and senior managers on macro-level issues of traffic accident prevention (policies, rules, and regulations, culture, etc.).
- ➢ Application of qualitative studies in designing, implementing, and evaluating RTIs prevention interventions and policies.

## Supplementary Information

**Additional file 1.** Complete search strategy in PubMed databases for identifying the qualitative studies on Road Traffic Injuries (RTIs) published between2000 to 2019.

**Additional file 2.** Results of the quality appraisal of qualitative studies on Road Traffic Injuries (RTIs) published between2000 to 2019.

## Data Availability

Supplementary files are available in the journal website.

## Abbreviations

RTIs: Road Traffic Injuries
EAGLE: European Association for Grey Literature Exploitation
HMIC: Health Care Management Information Consortium
SIGLE: System for Information on Grey Literature in Europe
PRISMA: Preferred Reporting Items for Systematic Reviews and Meta-Analyses
CASP: Critical Appraisal Skills Program
LMICs: Low-and-middle income countries
PTSD: Post-Traumatic Stress Disorder
GDP: Gross Domestic Product
HC: Human Capital
WTP: Willingness To Pay
NHTSA: National Highway Traffic Safety Administration
WHO: World Health Organization
IDSP: Integrated Disease Surveillance Project
