# Supplementary material for: Meta-synthesis of qualitative evidence in road traffic injury prevention: a scoping review of qualitative studies (2000 to 2019)
Source: Arch Public Health. 2020 Nov 3;78:110. doi: 10.1186/s13690-020-00493-0 (PMC7607842; doi:10.1186/s13690-020-00493-0)
Supplement: Supplementary file 1 — Additional file 1. Complete search strategy in PubMed databases for identifying the qualitative studies on Road Traffic Injuries (RTIs) published between2000 to 2019. [file 13690_2020_493_MOESM1_ESM.docx]

| Additional file1: Complete search strategy in PubMed databases for identifying the qualitative studies on Road Traffic Injuries (RTIs) published between2000 to 2019 | | |
| --- | --- | --- |
| **Set** | **Strategy** | **Results** |
| **#1** | ((((((((((road[Title]) OR traffic[Title]) OR injury[Title]) OR accident[Title]) OR crash[Title]) OR collision[Title]) OR Motor Vehicles[Title]) OR motorcycle[Title]) OR pedestrians[Title]) OR car[Title]) OR automobile[Title] | 229989 |
| **#2** | ((((qualitative[Title/Abstract]) OR interview[Title/Abstract]) OR focus group discussion[Title/Abstract]) OR phenomenology[Title/Abstract]) OR grounded theory[Title/Abstract] | 298763 |
| **#3** | #1 AND #2 | 2962 |
| * Filters activated: Journal Article, Full text, Humans, English | | |
