# Supplementary material for: Meta-synthesis of qualitative evidence in road traffic injury prevention: a scoping review of qualitative studies (2000 to 2019)
Source: Arch Public Health. 2020 Nov 3;78:110. doi: 10.1186/s13690-020-00493-0 (PMC7607842; doi:10.1186/s13690-020-00493-0)
Supplement: Supplementary file 2 — Additional file 2. Results of the quality appraisal of qualitative studies on Road Traffic Injuries (RTIs) published between2000 to 2019. [file 13690_2020_493_MOESM2_ESM.docx]

| Additional file2: Characteristic of qualitative studies on Road Traffic Injuries (RTIs) published between2000 to 2019 | | | | | | | | | | | |
| --- | --- | --- | --- | --- | --- | --- | --- | --- | --- | --- | --- |
| Author/year | Screening Questions | | Detailed questions | | | | | | | | Score (8-24) |
|  | 1 | 2 | 3 | 4 | 5 | 6 | 7 | 8 | 9 | 10 |  |
| (Doohan and Saveman 2014) | Yes | Yes | Yes | Can’t tell | Yes | Can’t tell | Yes | Yes | Yes | Yes | 22 |
| (Pashaei Sabet, Norouzi Tabrizi et al. 2016) | Yes | Yes | Yes | Yes | Yes | Can’t tell | Yes | Yes | NO | NO | 19 |
| (Yadav and Shrestha 2017) | Yes | Yes | Yes | Yes | Yes | Yes | NO | Yes | NO | Yes | 20 |
| (Franzen, Bjornstig et al. 2006) | NO | NO | - | - | - | - | - | - | - | - | - |
| (Pashaei Sabet, Norouzi Tabrizi et al. 2014) | Yes | Yes | Yes | Can’t tell | Yes | Can’t tell | Yes | Yes | Yes | Yes | 22 |
| (Ghorashi, Kazemi et al. 2012) | Yes | Yes | Yes | Can’t tell | Yes | Yes | NO | Can’t tell | Yes | NO | 18 |
| (Batool, Carsten et al. 2012) | NO | Yes | - | - | - | - | - | - | - | - | - |
| (Christie, Ward et al. 2007) | Yes | Yes | Yes | Can’t tell | Yes | Can’t tell | NO | Yes | Yes | Yes | 20 |
| (Sanusi and Emmelin 2015) | Yes | Yes | Yes | Yes | Yes | Yes | Yes | Yes | Yes | Yes | 24 |
| (Tetali, Lakshmi et al. 2013) | Yes | Yes | Yes | Yes | Yes | Can’t tell | Yes | Yes | Yes | Yes | 23 |
| (Hashemiparast, Montazeri et al. 2017) | Yes | Yes | Yes | Yes | Yes | Yes | Yes | Yes | Yes | Yes | 24 |
| (Shams, Shojaeezadeh et al. 2010) | Yes | NO | - | - | - | - | - | - | - | - | - |
| (Zamani-Alavijeh, Niknami et al. 2010) | Yes | Yes | Yes | Yes | Yes | Yes | NO | Yes | Yes | Yes | 22 |
| (Khorasani-Zavareh, Khankeh et al. 2009) | Yes | Yes | Yes | Yes | Yes | Yes | Yes | Yes | Yes | Yes | 24 |
| (Haghparast-Bidgoli, Khankeh et al. 2013) | Yes | Yes | Yes | Yes | Yes | Yes | Yes | Yes | Yes | Yes | 24 |
| (Haghparast-Bidgoli, Hasselberg et al. 2010) | Yes | Yes | Yes | Yes | Yes | Yes | Yes | Yes | Yes | Yes | 24 |
| (Alinia, Khankeh et al. 2015) | Yes | Yes | Yes | Yes | Yes | Yes | Yes | Yes | Yes | Yes | 24 |
| (Razzaghi, Pourrajabi et al. 2017) | Yes | NO | - | - | - | - | - | - | - | - | - |
| (Hashemiparast, Negarandeh et al. 2017) | Yes | NO | - | - | - | - | - | - | - | - | - |
| (Perez-Nunez, Pelcastre-Villafuerte et al. 2012) | Yes | Yes | Yes | Yes | Yes | NO | Yes | Yes | NO | Yes | 20 |
| (Noori Hekmat, Dehnavie et al. 2015) | Yes | Yes | Yes | Yes | Yes | NO | NO | Yes | Yes | Yes | 20 |
| (Bazeli, Aryankhesal et al. 2017) | Yes | Yes | Yes | Yes | Yes | Yes | Yes | NO | Yes | NO | 20 |
| (Huicho, Adam et al. 2012) | Yes | NO | - | - | - | - | - | - | - | - | - |
| (Ramos, Diez et al. 2008) | Yes | Yes | Yes | Yes | Yes | Yes | Yes | Yes | Yes | Yes | 24 |
| (Soori, Ainy et al. 2015) | Yes | Yes | Yes | Yes | Yes | Can’t tell | NO | NO | Yes | Yes | 19 |
| (Trevino-Siller, Hijar et al. 2011) | Yes | NO | - | - | - | - | - | - | - | - | - |
| (Ainy, Soori et al. 2011) | Yes | Yes | Yes | Yes | Yes | Can’t tell | NO | NO | Yes | Yes | 19 |
| (Salari, Motevalian et al. 2017) | Yes | NO | - | - | - | - | - | - | - | - | - |
| (Patel, Vissoci et al. 2017) | Yes | Yes | Yes | Yes | Yes | Yes | Yes | Yes | Yes | Yes | 24 |
| (Teye-Kwadjo 2017) | Yes | Yes | Yes | Can’t tell | Yes | Can’t tell | Yes | Yes | Yes | Can’t tell | 21 |
| 1- Was there a clear statement of the aims of the research?, 2- Is a qualitative methodology appropriate?, 3- Was the research design appropriate to address the aims of the research?, 4- Was the recruitment strategy appropriate to the aims of the research?, 5- Was the data collected in a way that addressed the research issue?, 6- Has the relationship between researcher and participants been adequately considered?, 7- Have ethical issues been taken into consideration?, 8- Was the data analysis sufficiently rigorous?, 9- Is there a clear statement of findings?, 10- How valuable is the research? | | | | | | | | | | | |
